# Cohort Profile: The Iodine Status in Pregnancy and Offspring Health Cohort (ISPOHC)

**DOI:** 10.2188/jea.JE20240349

**Published:** 2025-09-05

**Authors:** Zhuo Sun, Huiting Yu, YiXian Li, Wei Lu, Zhengyuan Wang, Qi Song, Shupeng Mai, Zehuan Shi, Liping Shen, Wenqing Ma, Xin Cui, Chen Xin, Jiajie Zang

**Affiliations:** 1 Division of Health Risk Factors Monitoring and Control, Shanghai Municipal Center for Disease Control and Prevention, Shanghai, China; 2 Division of Vital Statistics, Institute of Health Information, Shanghai Municipal Center for Disease Control and Prevention, Shanghai, China; 3 School of Public Health, Shanghai University of Traditional Chinese Medicine, Shanghai, China; 4 Shanghai Health Statistics Center, Shanghai, China

**Keywords:** iodine status, pregnancy, offspring, cohort

## Abstract

The Iodine Status in Pregnancy and Offspring Health Cohort (ISPOHC) was initiated in Shanghai to address the need for a comprehensive and longitudinal study on iodine nutrition and its effects on maternal and offspring health. The findings based on the Shanghai population can serve as a reference for other megacities experiencing significant dietary changes simultaneously. ISPOHC utilized a stratified cluster random sampling design, enrolling 5,099 pregnant women from all 16 districts of Shanghai. The survey has been conducted in three phases. Data collected at different time points include health status, living habits, dietary intake, birth, feeding, early development, anthropometric measurements, and biomarkers, allowing for an in-depth evaluation of iodine nutrition’s impact on offspring development. Data were collected through a combination of questionnaires, home visits, anthropometric measurements, and biological sample collection. The integration of detailed food investigation and on-site weighing of household seasonings provides a more precise assessment of dietary iodine intake, particularly iodized salt consumption, distinguishing this study. The study has provided significant insights into the relationship between iodine nutrition during pregnancy and various health outcomes.

## WHY WAS THE COHORT SET UP?

Since the adoption of The Outline of China’s Plan in 2000, 94.2% of counties in China had reached the goal of eliminating iodine deficiency disorder (IDD)^1^ by the end of 2015. The strategy to eliminate IDD in China has relied on the universal iodization of salt, which has proven to be effective. Being megacity along the eastern coast of China, the population of Shanghai has been facing the risk of iodine deficiency. Several factors must be considered in assessing and measuring iodine nutrient levels and risk of iodine deficiency, and the iodine content of the region’s soil and water is particularly crucial. Generally, the iodine concentration range of 40–100 µg/L is recommended in water suitable for iodine.^2^ The median water iodine level of 2.85 µg/L in Shanghai indicates an area of iodine deficiency, highlighting the need for iodine monitoring and iodization measures.

IDD results in impaired intellectual development in fetuses and children and can lead to maternal miscarriages, fetal abnormalities, and deaths.^3^ Also, iodine deficiency in pregnant women may cause thyroid dysfunction, affecting maternal thyroid autoimmune problems. Further, a previous study found that iodine deficiency during pregnancy is related to lower serum thyroxine (T4) levels, higher thyroid-stimulating hormone (TSH) levels, and a higher rate of anti-thyroglobulin antibody (TgAb) positivity, which may indicate hypothyroidism or thyroid autoimmune diseases.^4^ This can result in an increased risk of neurodevelopment and thyroid problems in offspring.^5^ Furthermore, another study revealed that low urinary iodine concentrations (UIC) during pregnancy increased the risk of delayed adaptive development in their offspring at 18–24 months.^6^ In addition, studies have shown that the adverse effects caused by iodine deficiency may not be apparent until the child attends school, manifesting a noticeable lag in the disease process.^7^ In 2020, a long-term follow-up study in iodine-deficient areas revealed that iodine deficiency during pregnancy was associated with lower psychomotor development scores in school-age offspring.^8^ This emphasizes the importance of conducting long-term longitudinal follow-up studies to assess the impact of iodine deficiency during pregnancy on the health of offspring.

It is worth to be noticed that pregnant women in Shanghai have mild iodine deficiency (100–150 µg/L), according to the results of the annual provincial representative iodine monitoring program conducted in Shanghai, which assesses iodine nutrition levels for pregnant women and ensures that the iodine levels in table salt meet national standards.^9^ This observation suggests further research on iodine health in pregnant women. However, the iodine monitoring program neither provides insights into long-term health outcomes nor infers the underlying cause of the disease.

Several regions in China have initiated longitudinal cohort studies on iodine nutrition, still there is lack of a diverse and generalized population sample and a more comprehensive and long-term study design. Maternal and child cohorts in Beijing,^10^ Wuhan,^11^ Tianjin,^12^ and Liaoning^13^ were conducted to explore the association between the iodine nutritional status of mothers and adverse pregnancy outcomes, fetal growth, and neonatal health-related biomarkers. However, some were single-center studies or had small sample sizes, limiting the general applicability of the results.

Shanghai is the busiest megacity in the eastern region of China; the study based on the population of Shanghai can provide a reference for many regions facing great changes at the same time. At present, there is a lack of long-term longitudinal research on the iodine intake status of pregnant women and the health status of their offspring. To address this gap, the Iodine Status in Pregnancy and its impact on their Offspring Health Cohort (ISPOHC) in Shanghai was established in 2017, aiming to determine the level of iodine intake among pregnant women in Shanghai and to provide more insights into the correlation of the iodine nutritional level and health status of pregnant women the effects of iodine on the health outcomes of the future generations.

## WHO IS IN THE COHORT?

The study was approved by the Ethical Committee of the Shanghai Municipal Center for Disease Control and Prevention (SCDC) (EC No. 2017/13, approval date: 25 April 2017).

We obtained informed consent from all participants in written form, and all items in the informed consent form were explained in detail by a professional community public health physician at the stage of the subject’s first enrollment. This includes the purpose and content of the project, confidentiality, voluntariness, possible risks and discomforts, and expected benefits. Participants can choose to withdraw at any stage of the study.

**Figure 1.**
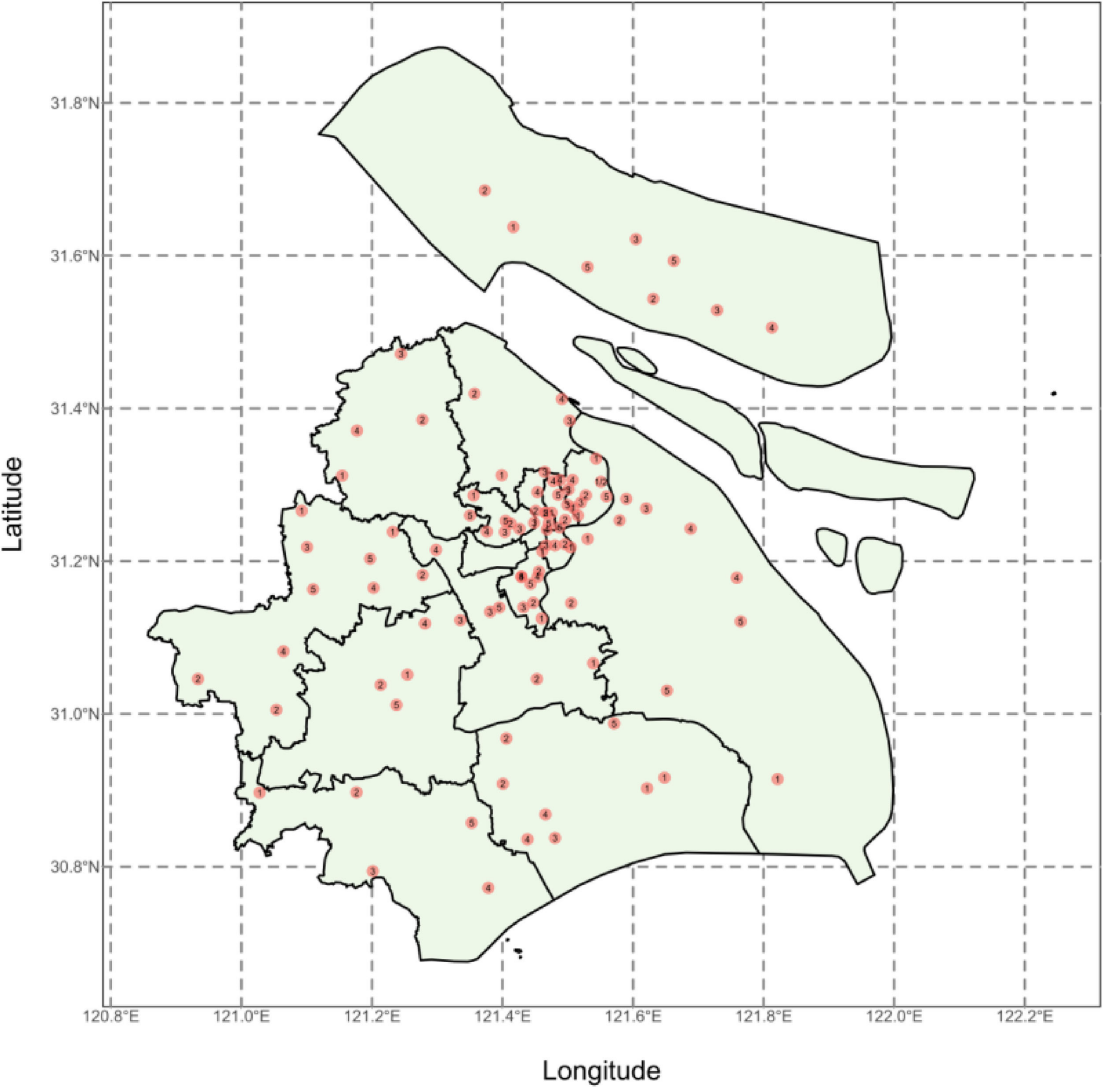
Distribution of the sampling areas of ISPOHC.

**Figure 2.**
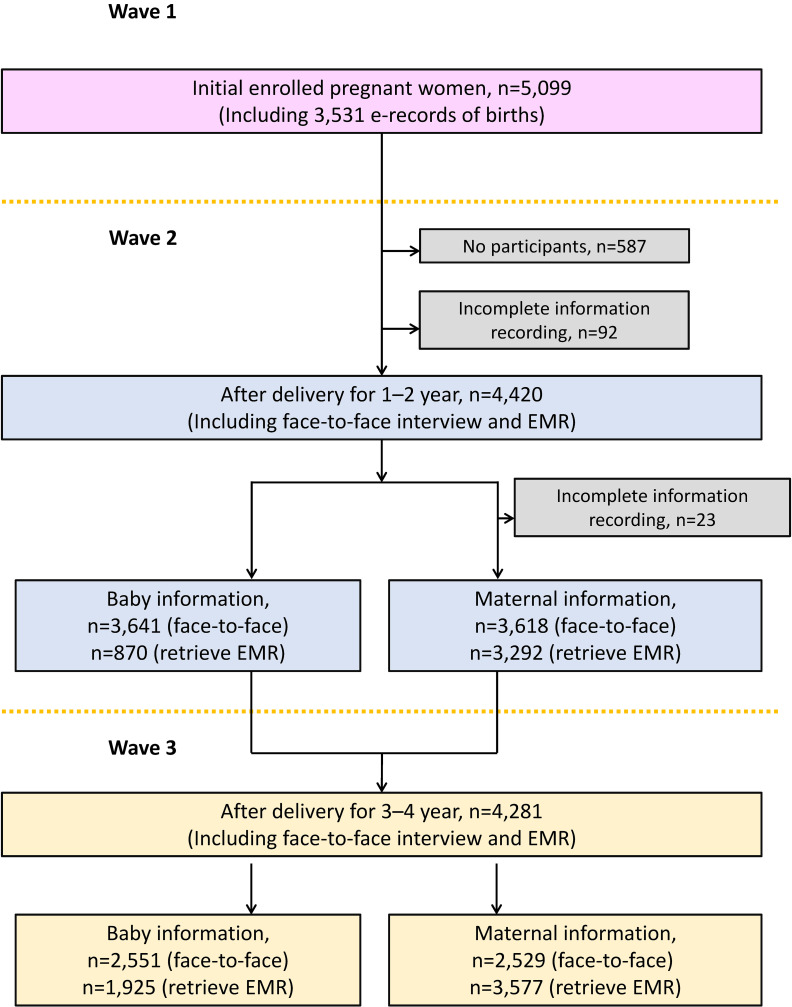
Flowchart illustrating the recruitment and exclusion processes of ISPOHC. EMR: Electronic Medical Records.

A representative sample of pregnant women newly enrolled in 2017 was selected using a stratified cluster random sampling design. All 16 districts in Shanghai were primary sampling units (PSUs), with each recruiting 350 participants, except Fengxian, Jinshan, and Chongming Districts, which recruited fewer due to lower birth rates. The distribution of the sampling areas of ISPOHC is demonstrated in Figure 1. Secondary sampling units (SSUs) were created by dividing each PSU into five sections and randomly selecting one street/town per section, or by using PSU maternal and child healthcare institutions. Recruitment began in April 2017 and concluded in April 2018. The study included 5,099 pregnant women, with 1,763 in the first, 1,791 in the second, and 1,545 in the third trimester. The recruitment and exclusion processes of ISPOHC are illustrated in Figure 2.

## HOW OFTEN HAVE THEY BEEN FOLLOWED UP?

**Figure 3.**
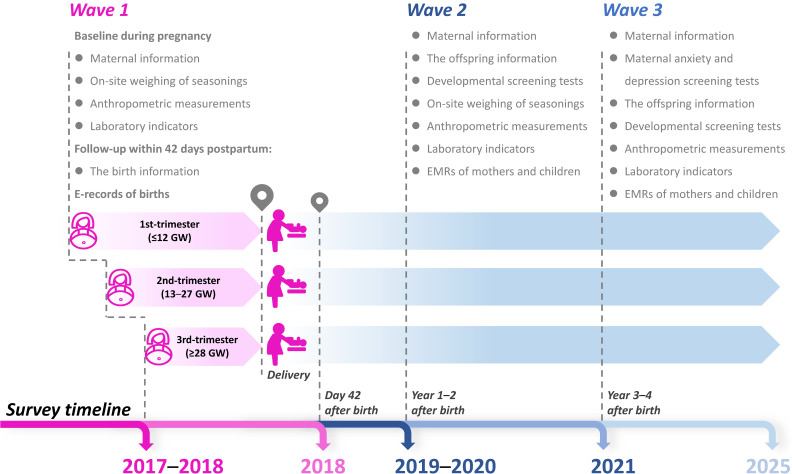
The schematic representation and schedule of ISPOHC

The ISPOHC survey has been conducted in three waves, with a fourth planned for 2025. The first wave (2017–2018) collected maternal and birth information, the second wave (2019–2020) focused on health behaviors and the health of mothers and children aged 1–2 years, and the third wave (2021–2022) updated information on children aged 3–4 years. The schematic representation and schedule of ISPOHC are demonstrated in Figure 3. Technical procedures for each survey were developed through prior study plans, internal discussions, and expert consultation. Before each survey, community physicians received training, and all waves included home visits, anthropometric measurements, and biological sample collection. Reliable birth registration and medical records were obtained in waves 2 and 3. Due to coronavirus disease 2019 (COVID-19), on-site weighing of seasonings was canceled in wave 3, replaced by online or telephone surveys. Quality control was strictly enforced by nutrition department staff at municipal and district centers, with specific personnel monitoring on-site activities and data accuracy.

## WHAT HAS BEEN MEASURED?

### Questionnaire framework

The survey was comprised of self-designed questions and international standardized scales. Table 1 presents information on the framework and specific items collected in every wave, and Table 2 illustrates the baseline data. Further, in the baseline survey during pregnancy, our questionnaire mainly focused on maternal demographic information, reproductive and disease history, and health risk factors. After the birth of the offspring (within 42 days postpartum), information on the delivery and birth outcomes was collected. As the offspring grew from 0 to 4 years, we designed questions for each follow-up, addressing different aspects.

**Table 1.** Framework and specific items collected in each wave

Research Framework	Measurements	Wave 1		Wave 2	Wave 3

		Baseline	Follow-up within 42 D		
**Maternal information**					
Demographics	Age, SES, marital status, birthplace, etc	✓		✓	✓
Lifestyle	Smoking, passive smoking, alcohol intake, physical activity, sitting time, sleeping time, etc	✓		✓	✓
Reproductive history and related behaviors	Menstrual history, obstetric history, obstetrical diseases history, etc	✓			✓^a^
Status of current pregnancy	Gestational week, pre-pregnancy weight, vaginal bleeding, lower abdominal pain, threatened miscarriage, gestational diabetes, gestational hypertension, thyroid abnormalities, etc	✓		✓^b^	
Fetal screening and intrauterine diagnosis	Down’s screening, deformity screening, placenta previa, oligohydramnios, malpresentation, fetal growth restriction, etc	✓		✓^b^	
Medical history of thyroid diseases	Thyroid enlargement, thyroid nodules, hyperthyroidism, hypothyroidism, Graves’ disease, Hashimoto’s thyroiditis, etc	✓		✓^a^	✓^a^
Medical history of metabolic diseases	Diabetes, hypertension, hyperlipidemia, hyperuricemia, etc			✓^a^	✓^a^
Occupational stress		✓		✓	✓
Postnatal depression	EPDS		✓		
Anxiety and depression screening test	CESD10 scale, self-rated anxiety SAS scale				✓
FFQ (during the past year)	The consumption frequency and types of salt and nutritional supplements; the consumption frequency and amount of common iodine-rich foods, staple foods, legumes, vegetables, mushrooms, algae, fruits, milk, nuts, meat, aquatic products, eggs, pickled products, tea, and coffee	✓		✓	✓
Additional eating habits	Eating out, SSBs intake				✓
**On-site weighing at home visits**					
Consumption of household seasoning	Initial and final weight of all seasonings during the 7 day investigation, the frequency and quantity of all family members dining at home within 7 days	✓		✓	
**Maternal anthropometric measurements**	Height, weight, abdominal circumference; hip circumference only in wave 3	✓		✓	✓
	Uterine height, fetal heartbeat	✓		✓	✓
	Blood pressure	✓			
	Grip strength				✓
**Maternal laboratory indicators**					
Fasting venous blood	Thyroid hormone (FT3, FT4, TSH, TT3, TT4) and 3 thyroid antibodies	✓		✓	
Random midstream urine	Urine creatinine, urine iodine, urine sodium	✓		✓	✓
Saliva sample					✓
Stool sample					✓
**The birth information**					
Birth information	Date of birth, child’s gender, birth length, birth weight, birth head circumference, Apgar score		✓		
Condition of delivery	Gestational age at delivery, mode of delivery, postpartum hemorrhage, puerperal infection, incision healing, postpartum urinary retention, maternal hospitalization duration, maternal post-delivery weight		✓		
**The offspring information**					
Demographics of child	Age, birthday, native place, gender, birth order, ethnicity			✓	✓
Knowledge and practice of feeding	Starting age, Onset of lactation, Breastfeeding challenges, Breastfeeding knowledge acquisition, Breastfeeding beliefs, duration, and frequency of exclusive breastfeeding/formula feeding/complementary feeding, usage of seasonings in baby food		✓	✓	
Feeding interactions and the dining habits	Child control, encourage/pressure for eating, involvement in family food selection; difficulty in feeding, the negative dining habits, feeding arrangements, feeding practices, child’s feeding reactions			✓	✓
FFQ for child (during the past month)	The consumption frequency and types of nutritional supplements; the consumption frequency and amount of common iodine-rich foods, staple foods, legumes, vegetables, fruits, dairy products, nuts, meat, aquatic products, eggs, animal innards, pickled products, cheese, and formula milk (in wave 2), snacks and fried food (in wave 3)			✓	✓
24 h food record for child					✓
Additional eating habits	Eating out, SSBs intake				✓
Family information	Primary caregiver information, SES of family, number of family members, number of siblings, consistency in family feeding concepts			✓	
Early development	Early education, age of speaking, the development of gross motor skills, postnatal cognitive stimulation, language development			✓	
Lifestyle	Sleep, physical activity, sedentary activity, and passive smoke exposure			✓	✓
Monitoring of growth	Monitoring frequency and method			✓	
Recent medical history	Respiratory disease, fever, diarrhea, asthma; clinical medication, etc			✓	✓
Oral and eye health	Dental caries, vision problems				✓
Food allergy history	Type and severity of food allergic			✓	✓
Clinical records	Blood routine examination			✓	
**Developmental screening tests**	DDST			✓	✓
**Child’s anthropometric measurements**	Body length/height, weight, head circumference, chest Circumference			✓	✓
	Blood pressure				✓
**Child’s laboratory indicators**					
Random midstream urine	Urine creatinine, urine iodine, urine sodium				✓
Saliva sample					✓
Stool sample					✓

CESD10 scale, Center for Epidemiological Studies Depression 10-item scale; DDST, Denver Development Screening Test. EPDS, Edinburgh Postnatal Depression Scale; FFQ, Food Frequency Questionnaire. SAS scale, Self-rating Anxiety Scale; SES, Socioeconomic Status; SSBs, Sugar Sweetened Beverages.

^a^The item is about the new developments since the last survey.

^b^The item is to recall the situation during the entire pregnancy period.

**Table 2.** Baseline characteristics of mothers-to-be participating in ISPOHC

	All participants	Different pregnancy stages *N* (%)		

	*N* (%)	First trimester	Second Trimester	Last Trimester
**Total**	5,099 (100)	1,763 (34.58)	1,791 (35.12)	1,545 (30.30)
**Age at delivery, years**				
<20	45 (0.88)	6 (0.34)	23 (1.28)	16 (1.04)
20–24	580 (11.37)	144 (8.17)	227 (12.67)	209 (13.53)
25–29	2,222 (43.58)	766 (43.45)	786 (43.89)	670 (43.37)
30–34	1,541 (30.22)	576 (32.67)	523 (29.20)	442 (28.61)
35–39	613 (12.02)	236 (13.39)	203 (11.33)	174 (11.26)
≥40	83 (1.63)	34 (1.93)	21 (1.17)	28 (1.81)
Not known	15 (0.29)	1 (0.06)	8 (0.45)	6 (0.39)
**Pre-pregnancy BMI**				
Underweight	647 (12.69)	250 (14.18)	225 (12.56)	172 (11.13)
Normal	3,508 (68.80)	1,192 (67.61)	1,239 (69.18)	1,077 (69.71)
Overweight	700 (13.73)	241 (13.67)	235 (13.12)	224 (14.50)
Obese	182 (3.57)	73 (4.14)	62 (3.46)	47 (3.04)
Not known	62 (1.22)	7 (0.40)	30 (1.68)	25 (1.62)
**Parity**				
0	3,047 (59.76)	1,110 (62.96)	1,041 (58.12)	896 (57.99)
1	1,875 (36.77)	620 (35.17)	696 (38.86)	559 (36.18)
2	168 (3.29)	32 (1.82)	53 (2.96)	83 (5.37)
≥3	9 (0.18)	1 (0.06)	1 (0.06)	7 (0.45)

**Demographic characteristics**				
**Educational background**				
Middle school and below	791 (15.51)	199 (11.29)	320 (17.87)	272 (17.61)
High school/Secondary vocational school/Technical school	802 (15.73)	229 (12.99)	312 (17.42)	261 (16.89)
Junior college/Specialized colleges	1,261 (24.73)	457 (25.92)	451 (25.18)	353 (22.85)
Undergraduate	1,876 (36.79)	723 (41.01)	597 (33.33)	556 (35.99)
Master	355 (6.96)	150 (8.51)	106 (5.92)	99 (6.41)
Others	2 (0.04)	0 (0.00)	1 (0.06)	1 (0.06)
Not known	12 (0.24)	5 (0.28)	4 (0.22)	3 (0.19)
**Partner’s educational background**				
Middle school and below	437 (8.57)	84 (4.76)	179 (9.99)	174 (11.26)
High school/Secondary vocational school/Technical school	811 (15.91)	244 (13.84)	285 (15.91)	282 (18.25)
Junior college/Specialized colleges	872 (17.10)	300 (17.02)	316 (17.64)	256 (16.57)
Undergraduate	1,535 (30.10)	563 (31.93)	506 (28.25)	466 (30.16)
Master	213 (4.18)	105 (5.96)	59 (3.29)	49 (3.17)
Not known	1,231 (24.14)	467 (26.49)	446 (24.90)	318 (20.58)
**Occupation**				
Clerical and related personnel	829 (16.26)	321 (18.21)	301 (16.81)	207 (13.40)
Commercial and service industry personnel	893 (17.51)	350 (19.85)	285 (15.91)	258 (16.70)
Professional and technical personnel	953 (18.69)	362 (20.53)	305 (17.03)	286 (18.51)
Stay at home	1,118 (21.93)	306 (17.36)	411 (22.95)	401 (25.95)
Others	1,278 (25.06)	414 (23.48)	478 (26.69)	386 (24.98)
Not known	28 (0.55)	10 (0.57)	11 (0.61)	7 (0.45)
**Marital status**				
Married or cohabiting	5,045 (98.94)	1,746 (99.04)	1,768 (98.72)	1,531 (99.09)
Others	39 (0.76)	13 (0.74)	17 (0.95)	9 (0.58)
Not known	15 (0.29)	4 (0.23)	6 (0.34)	5 (0.32)
**Family income in the last year**				
Under 99,000 CNY	885 (17.36)	240 (13.61)	329 (18.37)	316 (20.45)
100,000–199,000 CNY	2,074 (40.67)	705 (39.99)	759 (42.38)	610 (39.48)
200,000–349,000 CNY	1,520 (29.81)	572 (32.44)	485 (27.08)	463 (29.97)
350,000 CNY and above	600 (11.77)	239 (13.56)	209 (11.67)	152 (9.84)
Not known	20 (0.39)	7 (0.40)	9 (0.05)	4 (0.26)

**Lifestyle**				
**Smoking**				
Never smoke	4,950 (97.08)	1,704 (96.65)	1,735 (96.87)	1,511 (97.80)
Smokers	132 (2.59)	51 (2.89)	51 (2.85)	30 (1.94)
Not known	17 (0.33)	8 (0.45)	5 (0.28)	4 (0.26)
**Passive smoking**				
No	2,910 (57.07)	1,009 (57.23)	986 (55.05)	915 (59.22)
Yes	2,129 (41.75)	740 (41.97)	782 (43.66)	607 (39.29)
Not known	60 (1.18)	14 (0.79)	23 (1.28)	23 (1.49)
**Alcohol consumption**				
Pre-pregnancy drinkers				
No	4,553 (89.29)	1,576 (89.39)	1,586 (88.55)	1,391 (90.03)
Yes	512 (10.04)	175 (9.93)	194 (10.83)	143 (9.26)
Not known	34 (0.67)	12 (0.68)	11 (0.61)	11 (0.71)
Pregnant drinkers				
No	5,038 (98.80)	1,739 (98.64)	1,769 (98.77)	1,530 (99.03)
Yes	61 (1.20)	24 (1.36)	22 (1.23)	15 (0.97)

**Health condition**				
**Benign thyroid disease**				
No	4,508 (88.41)	1,553 (88.09)	1,565 (87.38)	1,390 (89.97)
Yes	556 (10.90)	201 (11.40)	212 (11.84)	143 (9.26)
Not known	35 (0.69)	9 (0.51)	14 (0.78)	12 (0.78)
**Benign breast diseases**				
No	3,692 (72.41)	1,278 (72.49)	1,280 (71.47)	1,134 (73.40)
Yes	1,321 (25.91)	460 (26.09)	468 (26.13)	393 (25.44)
Not known	86 (1.69)	25 (1.42)	43 (2.40)	18 (1.17)
**Benign gynecological diseases**				
No	3,917 (76.82)	1,316 (74.65)	1,384 (77.28)	1,217 (78.77)
Yes	1,079 (21.16)	420 (23.82)	362 (20.21)	297 (19.22)
Not known	103 (2.02)	27 (1.53)	45 (2.51)	31 (2.01)
**Uterine fibroid**				
No	4,860 (95.31)	1,646 (93.36)	1,712 (95.59)	1,502 (97.22)
Yes	239 (4.69)	117 (6.64)	79 (4.41)	43 (2.78)
**PCOS**				
No	1,190 (23.34)	451 (25.58)	416 (23.23)	323 (20.91)
Yes	121 (2.37)	60 (3.40)	29 (1.62)	32 (2.07)
Not known	3,788 (74.29)	1,252 (71.02)	1,346 (75.15)	1,190 (77.02)
**Hypertension**				
No	4,998 (98.02)	1,719 (97.50)	1,757 (98.10)	1,522 (98.51)
Yes	15 (0.29)	5 (0.28)	8 (0.45)	2 (0.13)
Not known	86 (1.69)	39 (2.21)	26 (1.45)	21 (1.36)
**Dysglycemia**				
No	5,041 (98.86)	1,745 (98.98)	1,768 (98.72)	1,528 (98.90)
Yes	29 (0.57)	9 (0.51)	10 (0.56)	10 (0.65)
Not known	29 (0.57)	9 (0.51)	13 (0.73)	7 (0.45)
**Hyperlipidemia**				
No	4,993 (97.92)	1,729 (98.07)	1,749 (97.65)	1,515 (98.06)
Yes	57 (1.12)	20 (1.13)	19 (1.06)	18 (1.17)
Not known	49 (0.96)	14 (0.79)	23 (1.28)	12 (0.78)

BMI, body mass index; PCOS, polycystic ovary syndrome.

### Demographics

The demographic information of the mother was self-reported, including her birth information, ethnicity, educational level, years of education, occupation, marital status, household population, total household income, and other relevant data. These items were developed concerning previous national monitoring questionnaires. The occupational classification in our questionnaire was based on *the Classification and codes of occupations* (GB/T 6565-2015).^14^ The classification of educational level refers to the national standard *Codes for records of formal schooling* (GB/T 46582006).^15^

### Lifestyle

The information regarding the status of maternal smoking was obtained through a series of questions designed to ask about personal smoking habits and secondhand smoke exposure. Alcohol intake before and during pregnancy was investigated separately by asking about the frequency and quantity of alcohol consumed each time. The physical activity level of participants was evaluated utilizing the full version of the International Physical Activity Questionnaire (IPAQ) in Chinese.^16^

### Medical history and clinical records

In wave 1, menstrual characteristics, gravidity, parity, adverse pregnancy outcomes, and the history of female-specific medical and surgical conditions were investigated. Additionally, mothers were asked to recall their medical history throughout pregnancy during the 42-day postpartum follow-up. In waves 2 and 3, inquiries focused on the child’s outpatient visits, medication records from the past 14 days, and detailed information on food allergies, including the types of food, age of occurrence, and severity.

### Maternal mental health assessment

The Chinese version of the Core Occupational Stress Scale (COSS) for occupational populations in China (COSS)^17^^,^^18^ and the Effort-reward Imbalance (ERI) Scale^19^ were used to assess maternal occupational stress in every wave.

The postpartum depression of the mother was evaluated via the Edinburgh Postnatal Depression Scale (EPDS),^20^ which includes a 10-item questionnaire, such as mood, pleasure, self-blame, anxiety, fear, insomnia, coping ability, sadness, crying, and self-injury. As the peak period of postpartum depression usually affects individuals within one month after delivery, the optimal time for EPDS screening in our study was determined to be 26 days after delivery.

### Diet

The food frequency questionnaire (FFQ), validated by SCDC, assessed the frequency of food and drink consumption. In waves 1 and 2, the FFQ included iodine-rich foods, staple foods, legumes, vegetables, fruits, milk, nuts, meat, aquatic products, eggs, pickled products, tea, and coffee. In wave 3, the FFQ added questions about sugar-sweetened beverages, snacks, fried foods, and barbecue foods, along with detailed records of nutritional supplements. Additionally, questions were designed to evaluate the long-term use and replacement of salt, focusing on the type and frequency of salt replacement.

### Birth information

We collected information on the date of birth, child’s gender, birth length, birth weight, birth head circumference, and Apgar score from the self-reported postnatal survey. In addition, data on the general condition of delivery, gestational age at delivery, delivery mode, postpartum hemorrhage, puerperal infection, wound healing, and postpartum urinary retention were also collected.

### Knowledge and practice of feeding

The questionnaires mainly focused on inquiring about knowledge and practice of infant feeding, especially breastfeeding, in wave 2, then conducted follow-up on these issues in wave 3. The items related to infant feeding were divided into two parts. The first part asked about mothers’ breastfeeding knowledge and practice, while the second part aimed to understand the current breastfeeding situation and complementary feeding of children.

### Feeding interactions and dining habits

Starting from wave 2, we have also included questions related to the feeding interactions of main caregivers and the child’s negative eating habits. The feeding interactions of the primary caregiver were reflected through eight items of four dimensions: child control, encouragement, pressure, and involvement. The negative dining habits of the child were reflected in five items.

### Child’s early development

Early childhood development was assessed through a self-designed structured questionnaire and the evaluation of gross motor skills using the Denver Development Screen Test II (DDSTII).^21^ Particularly, gross motor development was measured by the age at which the child could complete specific movements. DDSTII is a tool used to determine the developmental progress of infants and children aged 0–6 years. This test comprises 125 items across four sections, including personal social development, fine motor adaptive development, language development, and gross motor development. In addition, DDSTII also evaluates a child’s performance during the test, including compliance, interest in their surroundings, fear, and attention persistence.

### On-site weighing of household seasoning

The initial and final weights of main seasonings for family cooking were measured by community investigators on the 1^st^ and the 7^th^ day. These measurements were conducted after dinner on two consecutive Sundays. The main seasonings investigated include salt, oil, soy sauce, and other salty seasonings. Uniform electronic scales, accurate to 0.1 grams, were used for weighing. At the same time, the brand and type of seasoning (eg, whether the salt was iodized, whether the oil was animal/plant-based, and whether the soy sauce was re-sundried) were accurately recorded. The numbers of people dining at home were obtained through structural questions and were asked respectively by date and time.

### Anthropometric measurements

All physical measurements were carried out with participants wearing light clothing and no footwear. Each measurement was performed by experienced community physicians and was repeated and recorded at least twice. The body height (body length for the infant) and weight were measured using unified measuring instruments, with an accuracy of 0.1 cm and 0.1 kg, respectively. The circumference data (eg, uterine height, abdominal circumference, hip circumference) were taken with a flexible tape, with an accuracy of 0.1 cm. The blood pressure was measured on the individual’s right arm in a sitting position using the digital sphygmomanometer (OMRON HEM7071; OMRON Corporation, Kyoto, Japan) after a 15-minute rest. The hand’s Grip strength was measured using a digital hand dynamometer (CAMRY EH1101; Zhongshan Camry Electronic Co., Ltd., Zhongshan, Guangdong, China).

### Biological sample collection

For each participating mother, 10 mL venous blood samples were collected after 12 hours fasting in waves 1 and 2. The blood sample was drawn into two vacutainers, each containing 5 mL of heparin. After centrifuging, the top serum layer was separated into one 1 mL and six 0.5 mL tubes. Further, a random 20 mL midstream urine sample was collected from the mother during each wave and divided into five separate 4 mL tubes. In addition to a random midstream urine sample (20 mL), a saliva sample (23 mL), and feces sample (510 g) were also collected from the child in wave 3. After collection, the biological samples were kept at 4°C and transported to the designated laboratory for further subpackaging and testing. The samples were processed within 6 hours of collection and stored at −80°C for future use.

### Laboratory indicators

The concentrations of free triiodothyronine (FT3), free thyroxine (FT4), TSH, total triiodothyronine (TT3), total thyroxine (TT4), and three thyroid antibodies in serum were determined using electrochemiluminescence method employing automatic Access Immunoassay System (Siemens Healthcare Diagnostics Inc., Erlangen, Germany).

Furthermore, urine creatinine was determined using *Urine-Determination of creatinine-Spectrophotometric method* (WS/T 971996)^22^ or *Urine-Determination of creatinine-Reversed-phase high performance liquid chromatographic method* (WS/T 98-1996).^23^ Moreover, urine iodine and sodium were determined using *arsenic cerium catalytic spectrophotometric method* (WS/T 1072006)^24^ and classical clinical methods (eg, turbidimetry), respectively.

### E-record of mother and child

In wave 1, birth information can be rechecked through the birth registry system of SCDC, established in 2003 and provides authorized delivery services at all hospitals in Shanghai. The birth registry in Shanghai records all live births, excluding early pregnancy losses, abortions, or stillbirths, and includes information such as birth date, infant’s sex, weight, gestational age, congenital disabilities, and parental demographics. Congenital disabilities are diagnosed postnatally and coded according to ICD-10. In waves 2 and 3, electronic medical records (EMR) of mothers and children were retrieved from the Shanghai Health Statistics Center, including disease code, number of visits, and earliest visit time. The EMRs of maternal hypertension (including pregnancy-induced hypertension), eclampsia, abortion, diabetes (including pregnancy-induced diabetes), thyroid disease, anemia, intrauterine distress, and obesity, as well as for childhood inflammation, asthma, and infections, were obtained.

## WHAT HAS IT FOUND? KEY FINDINGS AND PUBLICATIONS?

To date, the following important topics have been discussed through ISPOHC.

### Assessment of iodine nutrition levels and iodized salt in pregnant women in Shanghai

Wang et al (2020) found that iodine level was adequate among pregnant women in Shanghai during the first and second trimesters, although it was insufficient in the third trimester.^25^ Good iodine-related knowledge, attitudes, and behaviors are important for pregnant women in maintaining satisfactory urinary iodine. After observing the dietary habits and patterns of pregnant women, Wang et al (2020) also found that household iodized salt did not play a decisive role in the iodine status of pregnant women. The current situation shows a low proportion of qualified-iodized salt used in home cooking. Still, foods eaten out have universal salt iodization according to the national compulsory policy, which indicates that pregnant women in their third trimester who are not eating out and using non-iodized salt at home require extra iodine.^26^

### Iodine nutrition during pregnancy and health outcomes

Chen et al (2022) found that inadequate iodine nutrition in pregnant women was an independent risk factor for thyroid autoimmunity in Shanghai.^27^ Hence, it is important to maintain adequate iodine status in pregnant women. Further, He et al (2020) found that state thyroid function during pregnancy could affect birth weight and outcome.^28^ However, the study conducted by Cui et al (2022) suggested that mild maternal iodine deficiency was not associated with adverse pregnancy outcomes.^29^

### Maternal dietary quality, patterns, and health outcomes

A significant association was found between specific dietary patterns and preterm birth.^30^ Further, it was determined that a higher ‘Animal Food Pattern’ (AFP) score was linked with a higher risk of preterm birth. The study concluded that a higher intake of AFP during pregnancy was positively associated with the risk of preterm birth. Furthermore, Wang et al discovered that adequate intakes of animal protein were associated with less likelihood of developing thyrotropin receptor antibody (TRAb) and a combination of thyroid peroxidase antibody (TPOAb), thyroglobulin antibodies (TgAb), and TRAb.^31^ This suggests that an adequate intake of animal protein during pregnancy protects against elevated levels of thyroid antibodies in pregnant women with mild iodine deficiency.

### The interactive effects between iodine nutrition and other factors

He et al (2019) observed seven urinary phthalate metabolites (PAEs) in >95% of pregnant women, with MnBP (geometric mean: 25.29 ng/mL) and MiBP (geometric mean: 11.18 ng/mL) being the most common PAEs detected.^32^ The positive association between edible seaweed intake and urinary MEP, MiBP, and DEHP levels was found after adjusting for covariates.

Lu et al (2022) reported that severe vitamin D deficiency combined with excess intake of iodine could increase the risk of TRAb positivity in pregnant women in the first trimester.^33^

Lu et al (2023) also found a positive trend in the cumulative effects of bisphenols (BPs) and iodine on serum FT3 and FT4, along with a U-shaped dose-response relationship between BPs and the probability of TPOAb+ in women with low UIC.^34^ There are some reported adverse health effects on the thyroid after co-exposure to BPs and iodine. Even though the pregnant women were exposed to lower levels of BPs, the women with iodine deficiency remained vulnerable to thyroid autoimmune disease.

## WHAT ARE THE MAIN STRENGTHS AND WEAKNESSES?

### Main strengths

The ISPOHC study has a comprehensive and prospective design focusing on iodine nutrition and its impact on pregnant women in Shanghai. We collect the data on iodine nutrition levels, pregnancy outcomes, and health status of pregnant women, which fills a significant gap in the region’s maternal and child health monitoring data. Using a stratified cluster random sampling design, the study included a representative cohort from all 16 districts, covering urban, suburban, and rural areas, allowing for detailed analysis across socioeconomic groups. Data collected spans health status, living habits, dietary intake, physical activity, and mental health, enabling a thorough assessment of iodine nutrition’s impact on offspring development. The study employed a combination of in-home weighing and FFQ for accurate dietary intake estimates, particularly iodine and other nutrients, establishing correlations between the intake of these nutrients and the growth and development of the offspring. Together with other data collected in the study, we could also explore the existing interactive effect. Advanced electronic data management systems were introduced to track all possible outcome events. This ensures that any common or uncommon health issues can be investigated in the future.

### Main weaknesses

A major challenge for the ISPOHC study is maintaining a low dropout rate due to the extended follow-up from early pregnancy to postpartum. The initial enrollment included some non-native women, who are more likely to leave Shanghai, potentially increasing dropout rates. To ensure data integrity and retention, the research team employed enhanced resource management and participant retention strategies, including external electronic data capture systems for outpatient and hospitalization records. During the COVID-19 pandemic, data collection methods were adapted by replacing face-to-face visits with online questionnaires or telephone surveys, minimizing inconvenience and risk for participants.

## CAN I GET HOLD OF THE DATA? WHERE CAN I FIND OUT MORE?

All ISPOHC data are held and managed by the research group at SCDC. There is an application process for using the data. After the application is approved by the Publication Committee, de-identified data can be shared with collaborators for research purposes. Discussion on potential collaboration can be requested by contacting the study investigators, Jiajie Zang, at the Shanghai Municipal Center for Disease Control and Prevention, China. Email: zangjiajie@scdc.sh.cn.

## References

[1] Wu Y, Li X, Chang S, Liu L, Zou S, Hipgrave DB. Variable iodine intake persists in the context of universal salt iodization in China. J Nutr. Sep 2012;142(9):1728–1734. 10.3945/jn.112.157982 22810983 PMC3417834

[2] Zhao W, Han C, Shi X, . Prevalence of goiter and thyroid nodules before and after Implementation of the universal salt iodization program in mainland China from 1985 to 2014: a systematic review and meta-analysis. PLoS ONE. Oct 2014;9(10):e109549. 10.1371/journal.pone.0109549 25313993 PMC4196906

[3] Li Y, Teng D, Ba J, . Efficacy and safety of long-term universal salt iodization on thyroid disorders: epidemiological evidence from 31 provinces of mainland China. Thyroid. Apr 2020;30(4):568–579. 10.1089/thy.2019.0067 32075540

[4] Chen W, Wang W, Gao M, . Iodine intakes of <150 µg/day or >550 µg/day are not recommended during pregnancy: a balance study. J Nutr. 2023;153(7):2041–2050. 10.1016/j.tjnut.2022.10.017 37100687

[5] Wu W, Chen Y, Guo W, . The relationship between iodine excess and thyroid function during pregnancy and infantile neurodevelopment at 18–24 months. J Nutr. 2023;153(8):2320–2327. 10.1016/j.tjnut.2023.05.012 37182695

[6] Wang K, Zhang J, Li F, . Urinary iodine in early pregnancy is associated with subclinical hypothyroidism in Tianjin, China: an observational study. BMC Endocr Disord. 2017;17(1):10. 10.1186/s12902-017-0162-x 28212640 PMC5316165

[7] Pearce EN, Lazarus JH, Moreno-Reyes R, Zimmermann MB. Consequences of iodine deficiency and excess in pregnant women: an overview of current knowns and unknowns. Am J Clin Nutr. 2016;104(Suppl 3):918S–923S. 10.3945/ajcn.115.110429 27534632 PMC5004501

[8] Toloza FJK, Motahari H, Maraka S. Consequences of severe iodine deficiency in pregnancy: evidence in humans. Front Endocrinol. 2020;11:409. 10.3389/fendo.2020.00409 32636808 PMC7318882

[9] Yang L, Li M, Liu X, . Evaluation of iodine nutritional status among pregnant women in China. Thyroid. Mar 2020;30(3):443–450. 10.1089/thy.2019.0001 31964276

[10] Zhang X, Yuan N, Sun J, . Association between iodine nutritional status and adverse pregnancy outcomes in Beijing, China: a single-center cohort study. Biol Trace Elem Res. Jun 2022;200(6):2620–2628. 10.1007/s12011-021-02887-9 34570342 PMC9132840

[11] Chen R, Li Q, Cui W, . Maternal iodine insufficiency and excess are associated with adverse effects on fetal growth: a prospective cohort study in Wuhan, China. J Nutr. Nov 2018;148(11):1814–1820. 10.1093/jn/nxy182 30239801

[12] Zhang Y, Du C, Wang W, . Effect of maternal and neonatal factors on neonatal thyroid stimulating hormone: results from a population-based prospective cohort study in China. J Trace Elem Med Biol. 2018;49:151–156. 10.1016/j.jtemb.2018.05.008 29895366

[13] Xiao Y, Sun H, Li C, . Effect of iodine nutrition on pregnancy outcomes in an iodine-sufficient area in China. Biol Trace Elem Res. 2018;182(2):231–237. 10.1007/s12011-017-1101-4 28770411

[14] China National Institute of Standardization. Classification and codes of occupations [GB/T 6565-2015].

[15] China National Institute of Standardization. Codes for record of formal schooling [GB/T 4658-2006].

[16] Macfarlane D, Lee CYC. Validity and reliability of the IPAQ (Chinese version - short form). Med Sci Sports Exerc. May 2004;36(5):S112. 10.1097/00005768-200405001-00529

[17] China Health Supervision Association. Guidelines of promotion for mental health in occupational groups (T/WSJD 13-2020). 2020.

[18] Wang J, Zhang QY, Chen HQ, . Development of the core occupational stress scale for occupational populations in China. Zhonghua Yu Fang Yi Xue Za Zhi. Nov 6 2020;54(11):1184–1189. 10.3760/cma.j.cn112150-20200319-00383 33147914

[19] Li XY, Guo YS, Zhang Y. Comment on “the reliability and validity of the effort-reward imbalance - the Chinese version”. Zhonghua Liu Xing Bing Xue Za Zhi. Jan 2006;27(1):25–28. 16737567

[20] Cox JL, Holden JM, Sagovsky R. Detection of postnatal depression. Development of the 10-item Edinburgh Postnatal Depression Scale. Br J Psychiatry. 1987;150:782–786. 10.1192/bjp.150.6.782 3651732

[21] Frankenburg WK, Dodds J, Archer P, Shapiro H, Bresnick B. The Denver II: A major revision and restandardization of the denver developmental screening-test. Pediatrics. Jan 1992;89(1):91–97. 10.1542/peds.89.1.91 1370185

[22] China National Institute of Standardization. Urine—Determination of creatinine—Part 1: Spectrophotometric method [WS/T 97-1996].

[23] China National Institute of Standardization. Urine—Determination of creatinine—Reversed-phase high performance liquid chromatographic method [WS/T 98-1996].

[24] China National Institute of Standardization. Urine—Determination of iodine—Part 1: Arsenic-cerium catalytic spectrophotometry [WS/T 107.1-2016].

[25] Wang Z, Wu Y, Shi Z, . Association of iodine-related knowledge, attitudes and behaviours with urinary iodine excretion in pregnant women with mild iodine deficiency. J Hum Nutr Diet. 2021;34(2):314–323. 10.1111/jhn.12837 33210387

[26] Wang Z, Jin W, Zhu Z, . Relationship of household cooking salt and eating out on iodine status of pregnant women in environmental iodine-deficient coastal areas of China. Br J Nutr. 2020;124(9):971–978. 10.1017/S000711452000207X 32517819

[27] Chen X, Wu C, Wang Z, . Iodine nutrition status and thyroid autoimmunity during pregnancy: a cross-sectional study of 4635 pregnant women. Nutr J. 2022;21(1):7. 10.1186/s12937-022-00760-6 35093086 PMC8801104

[28] He X, Yan Q, Liu C, . Association of maternal thyroid dysfunction and autoimmunity with adverse birth outcomes. Endocr Connect. 2022;11(4):e210599. 10.1530/EC-21-0599 35294399 PMC9066600

[29] Cui X, Yu H, Wang Z, . No association was found between mild iodine deficiency during pregnancy and pregnancy outcomes: a follow-up study based on a birth registry. Biol Trace Elem Res. 2022;200(10):4267–4277. 10.1007/s12011-021-03028-y 34988930 PMC9439975

[30] Wang Z, Zhao S, Cui X, . Effects of dietary patterns during pregnancy on preterm birth: a birth cohort study in Shanghai. Nutrients. 2021;13(7):2367. 10.3390/nu13072367 34371874 PMC8308829

[31] Wang Z, Shen J, Song Q, . Effects of animal protein intake during pregnancy on autoimmune thyroiditis in pregnant women with mild iodine deficiency. J Hum Nutr Diet. 2022;35(3):542–553. 10.1111/jhn.12954 34800315

[32] He X, Zang J, Liao P, . Distribution and dietary predictors of urinary phthalate metabolites among pregnant women in Shanghai, China. Int J Environ Res Public Health. 2019;16(8):1366. 10.3390/ijerph16081366 30995748 PMC6518169

[33] Lu W, Wang Z, Sun Z, . The interactive effects of severe vitamin D deficiency and iodine nutrition status on the risk of thyroid disorder in pregnant women. Nutrients. 2022;14(21):4484. 10.3390/nu14214484 36364747 PMC9654270

[34] Lu W, Sun Z, Wang Z, . The joint effects of bisphenols and iodine exposure on thyroid during pregnancy. Nutrients. 2023;15(15):3422. 10.3390/nu15153422 37571359 PMC10421451

